# Methods for investigating STAT3 regulation of lysosomal function in mammary epithelial cells

**DOI:** 10.1007/s10911-024-09563-3

**Published:** 2024-05-18

**Authors:** Bethan Lloyd-Lewis, Michael E. D’Angelo, Neve B. Prowting, Bethan E. Wiseman, Timothy J. Sargeant, Christine J. Watson

**Affiliations:** 1 School of Cellular and Molecular Medicine, Biomedical Sciences Building, University of Bristol, Bristol, BS8 1TD UK; 2 Biomedicine Discovery Institute, Monash University, Clayton, VIC 3800 Australia; 3 Hopwood Centre for Neurobiology, Lifelong Health Theme, Lysosomal Health in Ageing, South Australian Health and Medical Research Institute, Adelaide, Australia; 4 Department of Pathology, University of Cambridge, Tennis Court Road, Cambridge, CB2 1QP UK

**Keywords:** STAT3 signalling, Mammary gland, Cell death, Lysosome, Lysosome-mediated programmed cell death, Lysoptosis

## Abstract

The transcription factor STAT3 is activated by multiple cytokines and other extrinsic factors. It plays a key role in immune and inflammatory responses and, when dysregulated, in tumourigenesis. STAT3 is also an indispensable mediator of the cell death process that occurs during post-lactational regression of the mammary gland, one of the most dramatic examples of physiological cell death in adult mammals. During this involution of the gland, STAT3 powerfully enhances the lysosomal system to efficiently remove superfluous milk-producing mammary epithelial cells via a lysosomal-mediated programmed cell death pathway. The lysosome is a membrane-enclosed  cytoplasmic organelle that digests and recycles cellular waste, with an important role as a signalling centre that monitors cellular metabolism. Here, we describe key strategies for investigating the role of STAT3 in regulating lysosomal function using a mammary epithelial cell culture model system. These include protocols for lysosome enrichment and enzyme activity assays, in addition to microscopic analyses of the vesicular compartment in cell lines. Collectively, these approaches provide the tools to investigate multiple aspects of lysosome biogenesis and function, and to define both direct and indirect roles for STAT3.

## Introduction

Signal transducer and activator of transcription 3 (STAT3) is a transcription factor that is activated by multiple cytokines and other extrinsic factors to regulate a wide range of cellular processes. Our interest in STAT3 arose from early studies showing an association of high levels of phosphotyrosine STAT3 (pSTAT3) in extracts from mammary glands undergoing post-lactational regression (involution) [1, 2]. While pSTAT3 was undetectable in lactating mouse mammary glands, high levels were apparent within 12 h of forced weaning and remained high until day 6 of involution. Further work using mice in which STAT3 was conditionally deleted—using a milk protein gene promoter (β-lactoglobulin; BLG) to drive expression of Cre recombinase in milk-producing alveolar cells during lactation—showed that cell death during involution is extensively abrogated in the absence of STAT3 [3]. Although originally thought to be entirely mediated by apoptosis, this cell death process is first initiated by a lysosomal pathway (lysosomal-mediated programmed cell death, LM-PCD) [4–8] in which cathepsin proteases leak from lysosomes to bring about destruction of the cell.

Lysosomes are small, acidic, membrane-enclosed cytoplasmic organelles containing over 60 hydrolases that digest proteins, lipids, carbohydrates and nucleic acids, which are subsequently recycled to the cytosol. Integral lysosomal membrane proteins include the vacuolar (H +) ATPase that ensures the acidic pH of the lysosomal lumen, and heavily glycosylated proteins such as lysosomal-associated membrane proteins 1 and 2 (LAMP1 and LAMP2) that are protected on their cytosolic side from the acidic interior environment of the lysosome. The lysosomal membrane is also a platform for signalling complexes that sense and respond to changes in the cellular environment, including nutrient levels, energy availability and lysosome integrity. Lysosome biogenesis is regulated by the transcription factors TFEB and TFE3 in conditions of nutrient deprivation [9], while STAT3 regulates lysosome biogenesis in conditions of oxidative stress or substrate overload [10], discussed further below.

**Fig. 1:**
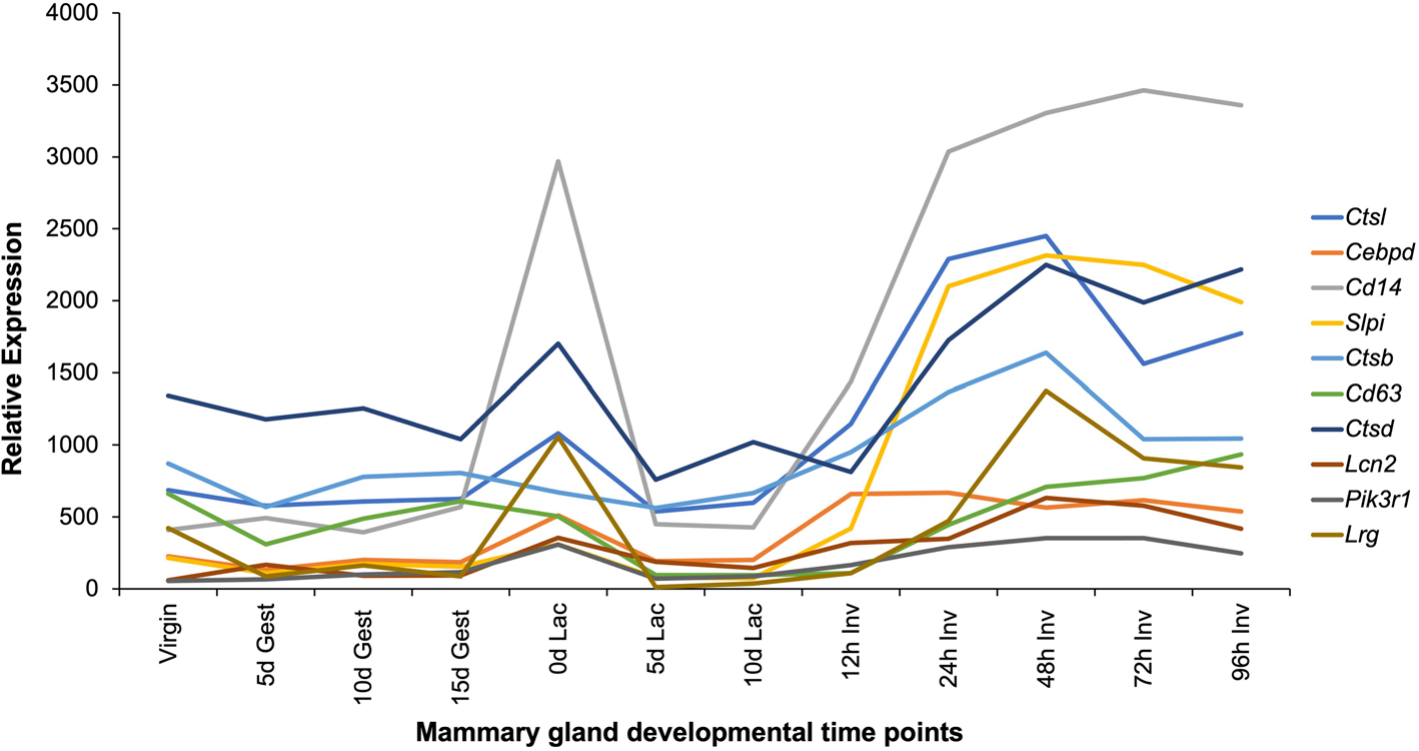
The distinctive gene expression profile of STAT3 target genes in mouse mammary tissues during pregnancy/lactation/involution. Profiles of selected genes from a microarray analysis of 12 time points during a murine mammary gland developmental cycle [12] showing the characteristic spike in expression at day 0 lactation followed by a dramatic increase during involution. The biological function of this lactation increase is not known but may be related to the sudden drop in progesterone at birth, and the switch to milk production. Some of these genes have been confirmed to be STAT targets either in vivo (*Cd14*, *Pik3r1, Slpi, Lrg1*) and/or in cell culture (*Cd63*) [14–17]

Microarray analysis of mouse mammary glands during a 6-day time course of involution identified a substantial number of genes that were either up- or down-regulated [11, 12], including a subset of STAT3 target genes [13]. The gene expression profile of many of these genes across 12 time points of pregnancy/lactation/involution is quite distinctive (Fig. 1), including the profiles for several lysosomal factors. Subsequently, our work has shown that STAT3 has a dual role in lysosome-mediated programmed death of secretory alveolar cells during involution: firstly, STAT3 activity dramatically downregulates the expression of an endogenous cathepsin inhibitor SPI2A (encoded by *Serpina3g*) that is highly expressed at the peak of lactation; and secondly, STAT3 upregulates the expression of a number of lysosomal peptidases, particularly cathepsin L and cathepsin B [4, 14]. Importantly, STAT3 also induces a cell fate switch during involution, whereupon secretory alveolar cells become phagocytic and take up milk fat globules and dying cells that have been shed into the alveolar lumen, increasing the demands on the lysosomal system [8]. These data suggested that STAT3 impacts on lysosomal function to mediate cell death during involution, a notion further supported by direct analysis of lysosomal integrity in both the presence and absence of STAT3 activity [4, 8].

To model STAT3 dependent LM-PCD in vitro*,* the murine mammary epithelial EpH4 cell line can be used. EpH4 cells are highly responsive to the cytokine oncostatin M (OSM), robustly activating pSTAT3 and inducing expression of lysosomal genes [4, 8, 15]. CRISPR/Cas9-deletion of STAT3 in EpH4 cells confirmed its role as a direct regulator of cathepsin B expression [15]. Interestingly, STAT3 also affects the glycosylation of the lysosomal membrane proteins LAMP1 and 2, both in vivo and in EpH4 cells [4, 15]. These data prompted us to investigate whether STAT3 affects not only lysosome biogenesis, but also lysosomal protein composition. To achieve this, we developed a method to enrich lysosomes from EpH4 cells using magnetic nanoparticles and conducted proteomic screens of lysosomal membrane proteins and assays to determine their ‘leakiness’ [8, 15].

We have previously provided detailed protocols for the analysis of STAT3 and lysosomes in mammary gland tissues [18]. Thus, in this article we describe methods that can be utilised to investigate STAT3 regulation of lysosomal function in vitro using the EpH4 cell line model of STAT3-dependent LM-PCD. We also provide a detailed protocol for the deletion of STAT3 using CRISPR/Cas9 in EpH4 cells. Other commonly used mammary epithelial cell lines should be amenable to these approaches. Although we have not tested our protocols on other cell types, we anticipate that they would be applicable without substantial modifications.

## Materials, reagents and solutions

### Cell culture and OSM stimulation of STAT3 activity

1. EpH4 cell line (*see* Note 1).
2. Dulbecco’s modified Eagle medium (DMEM, 11965092, ThermoFisher).
3. Fetal calf serum (FCS).
4. *Optional:* Penicillin–Streptomycin (15140122, ThermoFisher)
5. Cell culture flasks and dishes.
6. Trypsin–EDTA (0.05%, 25300104, ThermoFisher).
7. Phosphate-buffered saline (PBS).
8. Oncostatin M (OSM; 495-MO, R&D Systems): Made up in 0.1% (w/v) Bovine Serum Albumin (BSA) in PBS.
9. Primary and secondary antibodies to assess phospho-STAT3 levels by western blot (9131S, Cell Signalling. Suggested antibody dilution of 1:1000).

### Lysosome isolation using magnetic iron nanoparticles

1. Magnetic iron nanoparticles (EMG-508, Ferrotec).
2. 15 cm tissue culture plates.
3. Cell scrapers.
4. 1.5 ml microcentrifuge tubes.
5. PBS.
6. Centrifuge.
7. Handheld Dounce homogenizer.
8. Subcellular fractionation buffer: 20 mM HEPES–KOH pH 7.5, 250 mM sucrose, 10 mM KCL, 1.5 mM MgCl_2_, 1 mM EDTA, 1 mM EGTA, 8 mM dithiothreitol (DTT) and freshly added cOmplete Protease Inhibitor Cocktail (11697498001, Roche) (*see* Note 2).
9. Total cell extraction buffer: 0.1% (v/v) Triton X-100 (A4975, VWR) in PBS.
10. Lysosome membrane fractionation buffer: 5 mM Tris pH 7.5 in PBS.
11. Modified radioimmunoprecipitation (RIPA) buffer: 50 mM Tris–HCl pH 7.4, 1% (w/v) NP40, 0.25% (w/v) sodium deoxycholate, 150 mM NaCl, 1 mM EGTA in distilled water. Store at 4 °C. Prior to use, add cOmplete Protease Inhibitor Cocktail, 1 mM sodium orthovanadate (NaVO_3_, P0758L, NEB) and 50 mM sodium fluoride (NaF, 67414-1ML-F, Merck).
12. Magnetic microcentrifuge tube rack.

### Measuring lysosomal leakiness in response to STAT3 activation

#### Method 1: Cytosolic cathepsin activity assay

1. 6-well tissue culture plates.
2. Centrifuge.
3. Haemocytometer.
4. 1.5 ml microcentrifuge tubes.
5. Clear 96-well plates.
6. Digitonin (300410, Calbiochem).
7. 0.1% (v/v) Triton X-100 in PBS.
8. Subcellular fractionation buffer: 20 mM HEPES–KOH pH 7.5, 250 mM sucrose, 10 mM KCL, 1.5 mM MgCl_2_, 1 mM EDTA, 1 mM EGTA, 8 mM DTT, 1 mM Pefabloc (76307, Merck) (*see* Note 2).
9. Cathepsin reaction buffer: 50 mM sodium acetate, 8 mM EDTA, 8 mM DTT and 1 mM Pefabloc. Adjust pH to pH 6.
10. Synthetic cathepsin substrate Z-Phe-Arg-AMC (03–32-1501, Calbiochem).
11. Fluorescence plate reader.

#### Method 2: Lysosome leakiness assay using iron-nanoparticle isolated lysosomes

1. Subcellular fractionation buffer: 20 mM HEPES–KOH pH 7.5, 250 mM sucrose, 10 mM KCL, 1.5 mM MgCl_2_, 1 mM EDTA, 1 mM EGTA, 8 mM DTT and cOmplete Protease Inhibitor Cocktail (add fresh).
2. Modified radioimmunoprecipitation (RIPA) buffer: 50 mM Tris–HCl pH 7.4, 1% (w/v) NP40, 0.25% (w/v) sodium deoxycholate, 150 mM NaCl, 1 mM EGTA in distilled water. Store at 4 °C. Prior to use, add cOmplete Protease Inhibitor Cocktail, 1 mM sodium orthovanadate (NaVO_3_) and 50 mM sodium fluoride (NaF).
3. 1.5 ml microcentrifuge tubes.
4. Centrifuge.
5. Standard SDS PAGE and western blotting equipment.
6. Primary and secondary antibodies to detect LAMP2 (ab13524, Abcam), and Cathepsin B (ab214428, Abcam) and L (AF1515-SP, R&D) in fractions by western blot (suggested antibody dilution of 1:1000).

### CRISPR/Cas9 STAT3 KO cell line generation

1. HEK293T cells for lentivirus packaging.
2. EpH4 cells for targeting *STAT3.*
3. pLentiCRISPRv2 plasmid (52961, Addgene)
4. pCMVΔR8.91 packaging plasmid (V007548, NovoPro).
5. pMD2.G envelope plasmid (12259, Addgene).
6. Oligonucleotides for guide cloning.
7. DNA cloning enzymes (BsmBI, T4 polynucleotide kinase, T4 DNA ligase (NEB)).
8. E.coli for transformation (e.g. strains such as Stbl3 or NEBstable).
9. LB broth and agar plates containing 100 µg/ml ampicillin.
10. Polybrene (R-1003-G, Merck).
11. OptiMEM (31985070, ThermoFisher).
12. Polyethylenimine (PEI) 1 mg/ml in cell culture grade water as per manufacturer’s instructions (7854, Bio-techne).
13. 20 mM HEPES pH7.4, 150 mM NaCl in water, 0.22 µM filter sterilised (HBS).
14. Puromycin 10 mg/ml in sterile water (P8833, Merck).
15. 0.45 µm syringe filters.
16. 10 ml syringes.
17. 15 ml centrifuge tubes.
18. 50 ml centrifuge tubes.
19. 6 well tissue culture plates.
20. 10 cm tissue culture dishes.
21. 10% (v/v) FCS in DMEM.
22. Dimethyl sulfoxide (DMSO).
23. Mr Frosty freezing container (or equivalent).
24. Cryovials.
25. Genomic DNA extraction kit (e.g. 69504, QIAGEN).
26. Thermostable DNA polymerase (e.g. M7841, Promega).
27. PCR clean-up kit (e.g. 28106, QIAGEN).
28. Primary and secondary antibodies to assess STAT3 knockdown by western blot (9312, Cell Signalling).
29. Standard SDS PAGE and western blotting equipment.
30. 1% (w/v) agarose in TAE gel.

### Microscopic analysis of the vesicular compartment in cell lines

#### Method 1: Immunofluorescence or LysoTracker staining

1. Glass coverslips (No. 1.5) or glass-bottomed imaging dishes (e.g. Ibidi).
2. 4% (v/v) paraformaldehyde in PBS (11400580, Fisher Scientific).
3. PBS.
4. 0.5% (v/v) TritonX-100 in PBS.
5. Normal goat serum (G9023, Sigma, or serum from the species the secondary antibody is raised).
6. Primary and secondary antibodies as required e.g. rat anti-LAMP2 antibody (GL2A7, ab13524, Abcam, 1:200), rat anti-LAMP1 (ID4B, Developmental Studies Hybridoma Bank, 1:200).
7. LysoTracker ® probe e.g. LysoTracker Red DND-99 (L7528, ThermoFisher) (*see* Note 3).
8. DAPI solution: 1 μg/ml DAPI (4’,6-diamidino-2-phenylindole) dilactate (D9564, Sigma) in PBS.
9. 50% (w/v) glycerol in PBS, or preferred mounting medium.
10. Nail varnish (if using an aqueous, non-hardening mounting medium).
11. Fluorescence confocal microscope (e.g. a Leica SP5 or SP8)

#### Method 2: Transmission Electron Microscopy (TEM)

1. Cell scrapers.
2. 10 cm cell culture plates.
3. 1.5 ml microcentrifuge tubes.
4. Centrifuge.
5. PBS.
6. Fixative solution: 1.25% (v/v) glutaraldehyde and 4% (v/v) paraformaldehyde in PBS (4.9 mM Na_2_HPO_4_, 1.2 mM KH_2_PO_4_, 145.5 mM NaCl, pH 7.2).
7. Sucrose solution: 4% (w/v) sucrose in PBS.
8. 2% (w/v) osmium tetroxide in water (EMS19152, ProSciTech) (*see* Note 4).
9. Ethanol solutions: 70%, 90% and 95% (v/v) ethanol in water and 100% ethanol.
10. Propylene oxide (20412, ProSciTech). (*see* Note 4).
11. Resin (Procure 812 embedding kit (C038, ProSciTech).
12. 1:1 Propylene oxide: resin solution.
13. Ultramicrotome (e.g. a Leica EM UC7).
14. Mesh copper grids (GCU-PD200H, ProSciTech).
15. 4% (w/v) aqueous uranyl acetate (C079, ProSciTech) in water (*see* Note 4).
16. Filter paper.
17. Petri dishes.
18. Reynolds’ lead citrate stain [19].
19. Sodium hydroxide pellets (e.g. 567530, Merck).
20. Embedding mould (EMS70902-CB, ProSciTech).
21. Transmission Electron Microscope (e.g. Tecnai G2 Spirit TWIN (FEI, USA)).

## Methods

The following protocols describe in vitro biochemical and microscopy-based approaches for investigating the role of STAT3 in regulating lysosomal biogenesis and function in the EpH4 mouse mammary epithelial cell line [20], which may be readily adapted for different cell lines responsive to cytokine-induced STAT3 activation.

### Cell culture and OSM stimulation of STAT3 activity in EpH4 cells

The EpH4 normal murine mammary epithelial cell line was originally derived from the mammary gland of a mid-pregnant BALB/c female mouse [20]. OSM stimulation of EpH4 cells results in STAT3 activation and cell death, mimicking STAT3 dependent LM-PCD of mammary epithelial cells during in vivo mammary gland involution [4, 8, 15, 16, 18]. Thus, this cell line serves as a useful in vitro model for investigating the role of STAT3 signalling in lysosomal function and LM-PCD.

#### Cell maintenance and passage

1. Maintain EpH4 cells in 10% (v/v) FCS in DMEM in cell culture flasks at 37 °C with 5% CO_2_. If preferred, 100 U/ml Penicillin and 100 U/ml streptomycin (v/v) may be added to the medium.
2. When cells reach confluency, wash twice with PBS, and incubate with Trypsin–EDTA at 37 °C, 5% CO_2_ until cells detach.
3. Add 4 volumes of 10% (v/v) FCS in DMEM to inactivate the trypsin.
4. Dilute cell suspension 1:10 – 1:20 in new flasks containing fresh media (e.g. if the total volume of the cell suspension is 10 ml, transfer 1 ml (1:10 split)) into a new flask and top up with fresh medium. Incubate at 37 °C with 5% CO_2_.
5. Passage cells following steps 2–4 when confluent. Typically, when cells are split into new flasks as a ratio of 1:10 – 1:20 they will require passaging 2–3 times a week.

#### Stimulation of STAT3 activity in EpH4 cells

1. Trypsinise cells as described above.
2. Seed cells in multi-well plates or cell culture dishes as required for specific assays. For assays performed in standard 6-well tissue culture plates, seed EpH4 cells at a density of 1.0 × 10^5^ cells/well. Incubate at 37 °C with 5% CO_2_.
3. Upon reaching 50% confluency (typically next day when seeding 1.0 × 10^5^ cells/well in a 6-well plate), stimulate cells with 25 ng/ml of OSM (or 0.1% BSA in PBS as a ‘vehicle’ control) in 1% (v/v) FCS in DMEM (*see* Note 5).
4. 48 h later, renew medium with fresh OSM in 1% (v/v) FCS in DMEM.
5. Analyse cells at different time points as necessary. We recommend analysing EpH4 cells 48—72 h after OSM stimulation, when cells are beginning to undergo programmed cell death, but enough remain for analysis. Immunoblotting with a phospho-STAT3 antibody may be used to validate STAT3 activation in response to OSM stimulation.

### Lysosome isolation using magnetic iron nanoparticles

**Fig. 2:**
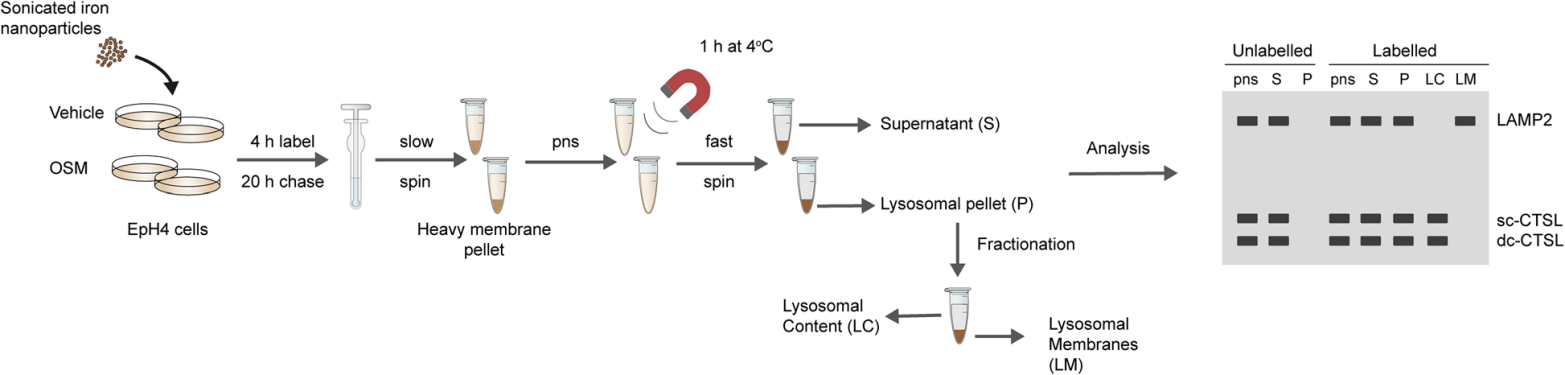
Isolation of EpH4 cell lysosomes using magnetic iron nanoparticles. Schematic representation of the magnetic iron nanoparticle lysosomal purification and fractionation protocol. Adapted from Lloyd-Lewis et al. (2018) [15]. Conditions have been optimised for isolating lysosomes from EpH4 cells to investigate the impact of STAT3 activation (via OSM stimulation) on the lysosomal compartment, which may be optimised or modified for different cell lines. For example, the efficiency of iron nanoparticle uptake can be cell-type dependent, and some cells may require a longer incubation time (provided cells remain healthy with longer iron exposures) or require more starting material to increase the yield of lysosomes isolated for downstream analysis. Isolated lysosomes can be analysed using a variety of downstream assays, including western blotting (illustrated), lysosomal enzyme activity assays or in lysosome leakiness assays. Alternatively, isolated lysosomes can be analysed by mass spectrometry or TEM

Conventional methods for isolating lysosomes revolve around using density centrifugation, often resulting in preparations contaminated by other organelles (e.g. mitochondria) of a similar density. Moreover, as lysosomes are heterogeneous in nature, variation in lysosomal buoyant density results in their distribution across a wide density gradient, hampering their purification using density centrifugation approaches. To overcome these difficulties, magnetic iron nanoparticles can be used. These nanoparticles are internalised by cells and delivered to lysosomes via the endocytic pathway, enabling their isolation by magnetic chromatography [8, 15, 21–23]. We have previously used this approach to isolate lysosomes from EpH4 cells to investigate the impact of OSM-induced STAT3 activation on lysosomal composition and permeability [8, 15]. Lysosomes isolated using magnetic iron nanoparticles can also be analysed by mass spectrometry, or by TEM [15]. This section describes the procedure for isolating lysosomes from EpH4 mammary epithelial cells using Ferrofluid iron nanoparticles (illustrated in Fig. 2), which can also be applied to other cell lines of interest.

#### Ferrofluid labelling of EpH4 cell lysosomes

1. Seed EpH4 cells at a density of 1 × 10^6^ cells in 15 cm cell culture dishes in 10% (v/v) FCS in DMEM. If not stimulating EpH4 cells with OSM, seed cells at a density of 3 × 10^6^ cells in 15 cm cell culture dishes and proceed directly to step 3 the next day (*see* Note 6).
2. Next day, treat cells with 25 ng/ml of OSM to stimulate STAT3 activity (as described above).
3. After 48 h of OSM stimulation, prepare the magnetic iron nanoparticles (Ferrofluid) by diluting 1:10 in 10 mg/ml BSA in PBS.
4. Sonicate the ferrofluid-BSA solution 3 times for 15 s on ice to disperse the iron nanoparticles. Rest samples for 15 s on ice between each sonication.
5. Filter-sterilise ferrofluid-BSA solution through a 0.2 µm filter attached to a syringe. Dilute the filter-sterilised ferrofluid-BSA solution 1:10 in 1% (v/v) FCS in DMEM.
6. Replace the media in the 15 cm dishes with the ferrofluid-containing DMEM and incubate for 4 h (‘label’ period).
7. Subsequently, wash EpH4 cells 3 times in PBS, and replace media with fresh 1% (v/v) FCS in DMEM and 25 ng/ml of OSM (or 0.1% (w/v) BSA in PBS as a ‘vehicle’ control) for a further 20 h (‘chase’ period).

#### Magnetic isolation of iron nanoparticle-containing lysosomes

8. Following the chase period (OSM stimulation time of 72 h), wash EpH4 cells 2 times in PBS.
9. Scrape cells in 1 ml of cold PBS and transfer to 1.5 ml microcentrifuge tubes.
10. Centrifuge at 900 × *g* for 3 min at 4 °C. Remove supernatant.
11. Add 700 µl of subcellular fractionation buffer containing fresh cOmplete Protease Inhibitor Cocktail (*see* Note 2) to cell pellets. Homogenise cells in a tight-fitting Dounce homogenizer with five strokes.
12. To remove nuclei and debris, spin the homogenate at 800 × *g* for 10 min at 4 °C. Transfer the supernatant to a clean 1.5 ml microcentrifuge tube and spin again at 800 × *g* for 10 min at 4 °C to ensure complete removal of contaminating heavy membranes.
13. Transfer the resulting supernatant (PNS; post nuclear supernatant) to a clean 1.5 ml microcentrifuge tube and load onto a magnetic rack (retain 50 µl for analysis by immunoblotting if required). Incubate for 1 h at 4** °**C on a rocker.
14. Following incubation, leave tubes on the magnet while removing the supernatant (abbreviated as S). Retain 50 µl for analysis.
15. Wash tubes attached to the magnetic rack 3 times with 1 ml of cold subcellular fractionation buffer. After the last wash, remove tubes from the magnetic rack and spin at 14,000 × *g* for 15 min at 4 °C to pellet magnetite-containing lysosomes.
16. Resuspend and combine pellets in a final volume of 200 µl of subcellular fractionation buffer and transfer to a new tube (‘whole lysosomes’) to remove any contaminating proteins that may have bound non-specifically to the side of the original tube. Pellet whole lysosomes, PNS and S samples by centrifuging at 14,000 × *g* for 15 min at 4 °C.
17. To extract protein lysates from the PNS and S samples, add 50 µl of modified RIPA buffer to pelleted material and incubate for at least 10 min on ice. To obtain “total” lysosomal protein lysates, incubate the pelleted ‘whole lysosomes’ fraction in 0.1% (v/v) Triton X-100 in PBS for 10 min on ice, intermittently vortexing every 2.5 min.
18. Subsequently spin all samples at 17,000 × *g* for 10 min at 4 °C and transfer the supernatant into clean 1.5 ml microcentrifuge tubes for downstream analysis (e.g. by immunoblotting, lysosomal leakiness assays as described in the subsequent section on measuring lysosomal leakiness). Alternatively, to separate lysosomal content and membrane fractions, continue as described below for fractionation of purified lysosomes.

#### Fractionation of purified lysosomes (optional)

19. If necessary to separate the lysosomal content from the lysosomal membrane fraction, resuspend the ‘whole lysosome’ fraction (isolated in Step 16) in lysosome membrane fractionation buffer and incubate on ice for 30 min. Alternatively, to improve lysosomal rupture, freeze samples in liquid nitrogen and thaw at 37 °C five times.
20. Pellet lysosomal membranes by spinning at 15,000 × *g* for 30 min at 4 °C.
21. Transfer the supernatant (lysosomal content (LC) fraction) to a new tube.
22. Resuspend the remaining pellet in 50 μl of 0.1% (v/v) Triton X-100 in PBS, incubate for 10 min on ice, intermittently vortexing four times.
23. Centrifuge tubes at 17,000 × *g* for 10 min at 4 °C. Transfer the supernatant (lysosomal membrane (LM) fraction) to fresh tubes.
24. Lysates obtained from total lysosome fractions (obtained in Step 17 in ‘Magnetic isolation of iron nanoparticle-containing lysosomes’ protocol) or LC and LM sub-fractions can be further analysed by immunoblotting or mass spectrometry analyses [15] (*see* Note 7).

### Measuring lysosomal leakiness in response to STAT3 activation

OSM treatment of EpH4 cells results in lysosomal membrane permeabilisation, leading to the leakage of lysosomal hydrolases into the cytosol [4, 8]. To measure the degree of lysosomal leakage, the activity of lysosomal cysteine cathepsins in the cytosolic compartment of EpH4 cells can be assessed using a sensitive enzyme activity assay [18, 24]. Cytosolic preparations can be obtained using a buffer containing the glycosidic detergent digitonin at a concentration that enables the extraction of cytosolic proteins from EpH4 cells without damaging lysosomal membranes. As OSM treatment also increases cathepsin protein expression in EpH4 cells, cathepsin activity in the cytosolic extraction must be normalised to total activity, which can be measured in total cell extracts obtained using 0.1% (v/v) Triton X-100 in PBS. Subsequently, a kinetic assay based on the cleavage of the fluorescent molecule AMC from the synthetic substrate Z-Phe-Arg-AMC by lysosomal cysteine cathepsins can be used to assess cytosolic cathepsin activity over time. The initial rate of fluorescence (corresponding to the initial rate of cathepsin activity (fluorescence/min)) is subsequently determined from the linear part of the resulting curve [18, 24]. Alternatively, lysosomes can be isolated directly using the purification method described above for in vitro leakage assays.

#### Method 1: Assessing lysosomal leakiness by measuring cytosolic cathepsin activity

1. Seed EpH4 cells at a density of 1.0 × 10^5^ cells/well in a 6-well tissue culture plate in 10% (v/v) FCS in DMEM. Next day, stimulate STAT3 activity using OSM as described above.
2. At the desired timepoint after OSM stimulation, remove media and wash cells in PBS. Incubate cells in Trypsin–EDTA until detached. Inactivate trypsin in 10% (v/v) FCS in DMEM and transfer samples to 15 ml centrifuge tubes.
3. Centrifuge samples at 200 × *g* for 5 min. Resuspend cell pellets in 1–2 ml of DMEM.
4. Count cells using a haemocytometer. Transfer 175,000 cells into two 1.5 ml centrifuge tubes per condition.
5. Centrifuge samples at 1000 × *g* for 3 min at 4 °C.
6. For each condition, resuspend cell pellets in 300 μl of fractionation buffer containing 25 μg/ml digitonin (for cytosolic extraction) or 0.1% (v/v) Triton X-100 in PBS (for total cell extraction).
7. Incubate cells on ice for 10 min. Pulse vortex samples every 2.5 min.
8. Centrifuge samples at 16,000 × *g* for 2 min at 4 °C.
9. Transfer supernatant into clean 1.5 ml tubes. For each condition there should be a digitonin and Triton X-100 extracted sample.
10. Per well of a 96 well plate (3 wells per sample), add 10 μl of the extracted sample, 160 μl of cathepsin reaction buffer and 30 μl of substrate (final concentration 50 μM) in reaction buffer (2 μl of 5 mM Z-Phe-Arg-AMC in 28 μl of cathepsin reaction buffer). In addition, prepare 3 wells of 200 μl of cathepsin reaction buffer only (‘Blank’ control), and 170 μl of cathepsin reaction buffer + 30 μl of substrate/reaction buffer (i.e. without sample, ‘Assay control’).
11. Measure fluorescence at 1 min intervals for 1 h at 37 °C in a fluorescent plate reader (excitation: 380 nm, emission: 442 nm).
12. Subtract background (mean of assay control wells) from all measurements and plot fluorescence over time for each sample. From the linear part of the resulting curve determine the initial rate of fluorescence for each sample corresponding to the initial rate of cathepsin activity (fluorescence/min).
13. Normalize cytosolic activity to total activity (measured in samples extracted with 0.1% (v/v) Triton X-100 in PBS). Typically, a 1.5-fold increase in cytosolic cathepsin activity is observed in EpH4 cells treated with OSM.

#### Method 2: Assessing lysosomal leakiness in isolated EpH4 cell lysosomes

**Fig. 3:**
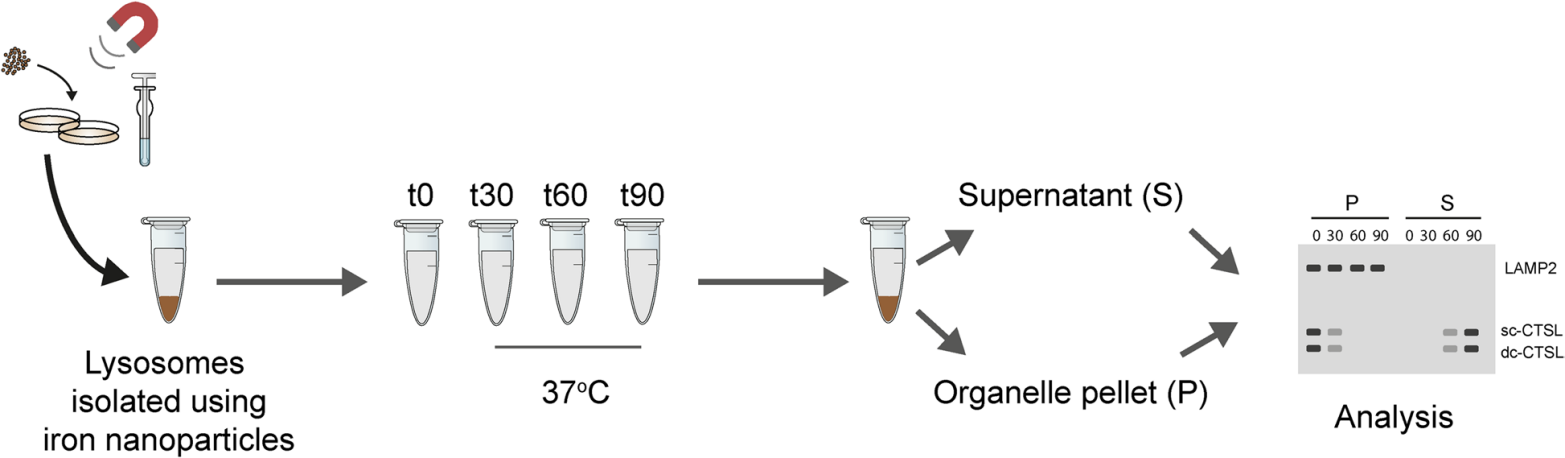
Schematic overview of the lysosomal leakiness assay. The permeability of isolated lysosomes can be assessed by western blotting for cathepsin B and L antibodies to visualise the degree of leakiness of cathepsin hydrolases into the supernatant fraction under different treatment conditions (i.e. vehicle and OSM stimulated conditions in EpH4 cells). Immunoblotting for LAMP2, a lysosomal membrane protein, can be used to standardise the amount of lysosomal material in a pellet during the assay. Alternatively, cathepsin activity levels can be assessed in supernatant and pellet fractions using enzyme activity assays

1. Isolate lysosomes from EpH4 cells stimulated with OSM to induce STAT3 activity (as described above).
2. After pelleting magnetite-containing lysosomes (Step 16 in ‘Magnetic isolation of iron nanoparticle-containing lysosomes’ protocol), resuspend in 200 µl of subcellular fractionation buffer and aliquot equal amounts (30 µl) into individual tubes (e.g. 4 tubes per treatment condition representing 0, 30, 60 and 90 min timepoints as depicted in Fig. 3).
3. Take one tube (per treatment condition) and centrifuge immediately at 14,000 × *g* for 15 min at 4 °C (time = 0 min). Carefully remove the supernatant without disturbing the pellet and snap freeze both the collected supernatant (*S0*) and pellet (*P0*). These fractions indicate the level of background leakage that occurs during processing.
4. Incubate other tubes at 37 °C with agitation for 30, 60, and 90 min respectively.
5. At the defined timepoints, centrifuge tubes at 14,000 × *g* for 15 min at 4 °C and carefully remove the supernatant without disturbing the pellet. Snap freeze both the collected supernatant (*S 30/60/90*) and pellet (*P 30/60/90*).
6. Resuspend the pellets (P fraction) in 150 μl of RIPA buffer (with protease inhibitors added fresh) and incubate for 30 min on ice with intermittent vortexing (3 pulses of 10 s). Centrifuge at 16,000 × *g* at 4 °C, for 15 min and remove the supernatant. S fractions from step 5 do not require further lysis.
7. For immunoblotting analysis, load equal volumes of the supernatant/pellet fractions per condition.
8. Probe western blots with cathepsin B and L antibodies to visualise the degree of leakiness of cathepsin hydrolases into the supernatant fraction under different treatment conditions (i.e. vehicle and OSM stimulated conditions). Immunoblotting with a LAMP2 antibody can be used to validate lysosomal integrity during the assay, in addition to standardising the amount of lysosomal material in a pellet during the assay.

### CRISPR/Cas9 mediated deletion of STAT3 in cell lines

The CRISPR/Cas9 system from *Streptococcus pyogenes* is the most widely adopted genome editing technology in use today due to its ease of use, high efficiency, and versatility of formats. Like many cell lines, EpH4 cells are highly refractory to transfection using lipofection reagents, but are readily transduced with lentivirus, which we implemented for CRISPR reagent delivery. We chose to use pLentiCRISPRv2 as it provides an all-in-one Cas9 and single guide RNA (sgRNA) expression system and allows for positive selection of transduced cells using puromycin. When a high efficiency guide is selected, the resulting pool of cells are often virtually a complete knockout, with no protein detectable by western blot. This is particularly important for epithelial cell lines such as EpH4 cells, where single cell cloning is best avoided as derived clones are likely to possess an altered phenotype from adapting to growth without normal epithelial cell-to-cell attachment and signalling. As the pool of cells transduced with pLentiCRISPRv2 constitutively express Cas9 and the sgRNA, the potential for cutting the genomic DNA at unintended loci (off-target sites) is increased. Therefore, a number of controls are essential when using all-in-one CRISPR lentivirus: 1) at least 2 independent pools of knockout cells should be established using different guide sequences that target the same gene (as any off-targeting cutting sites will not be shared); 2) a pool of cells targeted with a guide that does not have any matches in the genome (non-targeting guide) to control for any effects of lentiviral transduction, Cas9 expression and puromycin selection; and 3) a non-transduced “wild-type” control pool. To minimise the time for off-target cutting to accumulate, transduced cells should be cultured for the shortest number of passages as possible post-transduction (*see* Note 8). When designing guide sequences to target the gene of interest, use a tool such as that published by Doench et al. [25] to select for guides with a predicted high cutting efficiency (high on-target score) and low chance of cutting at other sites (low off-target score). Suggested guides for targeting mouse *STAT3* and a non-targeting control guide are provided in Table 1.

**Table 1:** Primer sequences for ‘CRISPR/Cas9 mediated deletion of STAT3 in cell lines’ protocol

Primer name	Sequence	Use	Notes
mStat3-CRISPR-F1	caccgGTACAGCGACAGCTTCCCCA	CRISPR	Anneal with mStat3-CRISPR-R1
mStat3-CRISPR-R1	aaacTGGGGAAGCTGTCGCTGTACc	CRISPR	Anneal with mStat3-CRISPR-F1
mStat3-CRISPR-F2	caccGGAACTGCCGCAGCTCCATG	CRISPR	Anneal with mStat3-CRISPR-R2
mStat3-CRISPR-R2	aaacCATGGAGCTGCGGCAGTTCC	CRISPR	Anneal with mStat3-CRISPR-F2
NTC_F1	caccgAAATTAAATTTAATTTAAAG	non-targeting control	Anneal with NTC_R1
NTC_R1	aaacCTTTAAATTAAATTTAATTTc	non-targeting control	Anneal with NTC_F1
hU6-fwd	GAGGGCCTATTTCCCATGATT	Sequencing	For sequencing guides cloned into pLentiCRISPRv2
STAT3-TIDE-F	GATCCCCAGCATCAGGAGTG	TIDE PCR	To amplify a 806 bp product from genomic DNA spanning the CRISPR cut sites in both STAT3KO pools. Use this product in TIDE assay
STAT3-TIDE-R	CACGCAGATAGGGCTGACTT	TIDE PCR	To amplify a 806 bp product from genomic DNA spanning the CRISPR cut sites in both STAT3KO pools. Use this product in TIDE assay

#### Cloning CRISPR guides into pLentiCRISPRv2

1. Prepare a batch of pLentiCRISPRv2 plasmid cut with the restriction enzyme BsmBI. Digest 2–3 µg of plasmid with 10 U of enzyme for 3 h at 55 °C. Gel extract the larger 12 kb fragment (using any commercial gel extraction spin column kit), avoiding the 2 kb filler sequence fragment. Quantify DNA concentration using a Nanodrop.
2. Phosphorylate and anneal the forward and reverse guide oligos by setting up the following 10 µl reaction: 1 µl of each oligo (each at 100 µM initial concentration), 6.5 µl of nuclease free water, 1 µl of 10X T4 ligase buffer and 0.5 µl of T4 DNA polynucleotide kinase. Place the reaction in a thermocycler and run the following cycle: 37 °C for 30 min, 95 °C for 5 min, then ramp down as slowly as possible (usually 5 °C per min) to 20 °C. Dilute the annealed oligos 1:200 in nuclease free water.
3. Ligate the annealed oligos into the cut plasmid in the following reaction: cut plasmid (20–50 ng), diluted annealed oligos (1 µl), 10X T4 DNA ligase buffer (1 µl), water to 9.5 µl and 0.5 µl of T4 DNA ligase added last. Prepare a control ligation with no annealed oligos (vector alone control). Incubate the ligation at room temperature for at least 1 h.
4. Transform a chemically competent RecA negative *E.coli* strain such as Stbl3 or NEBstable according to manufactures’ instructions. Spread on LB agar plates containing 100 µg/ml ampicillin and culture for 24 h at 30 °C. There should be at least tenfold more colonies on the vector + oligos plate than the vector alone plate.
5. Pick 2 colonies per guide, grow an overnight culture in LB containing 100 µg/ml of ampicillin for 16 h at 30 °C in a shaking incubator at approximately 250 r.p.m.
6. Prepare miniprep DNA using a standard spin-column based kit, and sequence using the hU6-fwd primer from Table 1.
7. For colonies containing a correct guide sequence, prepare a larger scale plasmid prep (midi or maxiprep) to obtain sufficient DNA for lentivirus packaging (at least 20 µg).

#### Packaging lentivirus for transduction (see Note 9)

1. Day 1:
  For each lentivirus to package, seed 1.5 x 10^6^ HEK293T cells in one 10 cm dish containing 10 ml 10% (v/v) FCS in DMEM. Culture cells at 37°C overnight.
2. Day 2:
  - In a 15 ml tube, prepare the plasmid transfection solution by mixing 18.25 µg of pLentiCRISPRv2 + guide plasmid, 5.84 µg of pMD2.G plasmid and 11.68 µg of pCMVΔR8.91 plasmid in 1.5 ml of HBS. Add 82 µl of PEI dropwise, then vortex vigorously for at least 10 s.
  - Incubate at room temperature for 10 min.
  - Add 4 ml of room temperature OptiMEM and mix by inverting the tube 6–8 times.
  - Incubate for a further 10 min at room temperature.
  - Aspirate media from cells and add the ~ 5.5 ml of transfection mixture and return to the incubator for 4 h.
  - Aspirate the transfection mixture and replace with 8 ml of 10% (v/v) FCS in DMEM and culture (see Note 9).
3. Day 4:
  - 48 h after transfection, collect the culture supernatant (containing lentivirus) from the HEK293T cells using a 10 ml syringe. Filter through a 0.45 µm filter into a 10 ml tube. This is the 48 h viral supernatant; use for transduction immediately (following protocol below), or store at -80 °C until required.
  - Add 8 ml of 10% (v/v) FCS in DMEM to the HEK293T cells and culture for a further 24 h to collect additional lentivirus.
4. Day 5: Collect the culture supernatant (containing lentivirus) from the HEK293T cells using a 10 ml syringe. Filter through a 0.45 µm filter into a 10 ml tube. This is the 72 h viral supernatant; use for transduction immediately (using protocol below) or store at -80 °C until required.

#### Transducing, selection, and cryopreservation of EpH4 cells (see Note 9)

1. The day before viral transduction, seed 1.0 × 10^5^ EpH4 cells per well of a 6-well tissue culture plate in 2 ml of 10% (v/v) FCS in DMEM. Include one extra well as a control for puromycin selection. Culture cells overnight.
2. Add polybrene at a final concentration of 4 µg/ml to the 48 h viral supernatant and mix well. 2 wells of a 6-well plate can be transduced with the viral supernatant from one 10 cm dish.
3. Aspirate media from EpH4 cells and add the viral supernatant/polybrene mixture. Culture cells overnight.
4. Next day, repeat steps 2–3 using the 72 h viral supernatant to increase efficiency of lentiviral transduction.
5. Transduced EpH4 cells will likely be confluent at this stage, so passage the cells as described above. Maintain two wells of a 6 well plate per transduction and one control well.
6. 24 h after passaging, add puromycin to a final concentration of approximately 1 µg/ml (optimal concentration should be determined empirically for each batch of puromycin and specific cell type).
7. Monitor the plate for cell death by observing cell detachment from the dish under a phase contrast microscope. When all the cells in the control well are dead (after approximately 48–72 h), the transduced cells should be > 90% viable, and confluent. Passage the cells, combining the 2 wells into a T175 tissue culture flask.
8. 48 h later, the flask should be 80–90% confluent. Cryopreserve several vials of cells for later use by trypsinising the cells, pelleting at 300 × *g* for 5 min, then resuspending in 10% (v/v) DMSO and 10% (v/v) FCS in DMEM at a concentration of 1–2 × 10^6^ cells per ml. Add 1 ml per cryovial, place inside a Mr. Frosty freezing container and leave at -80 °C overnight before transferring to liquid nitrogen for long-term storage. These cells are 3 passages post-transduction (*see* Note 8).

#### Assessing STAT3 deletion at the genomic DNA level using TIDE analysis

1. Prepare genomic DNA from 0.5–1.0 × 10^6^ cells from the STAT3 targeted cell lines and a wild-type control line.
2. Perform a PCR using the primers included in Table 1 using standard procedures for a commercial polymerase in a 50 µl reaction. Use 50 ng of genomic DNA as template.
3. Run 5 µl of the PCR product on a 1% (w/v) agarose in TAE gel and confirm the presence of a single product of the expected size (see Table 1).
4. Purify the remaining 45 µl of PCR product using a standard commercial PCR clean-up kit.
5. Submit samples for Sanger sequencing. Sequence with both the forward and reverse primer (Table 1).
6. Analyse the sequencing results using the TIDE web interface (http://shinyapps.datacurators.nl/tide/ (*see* Note 10)).
7. The software will provide a quantification of the level of indels found in the sample, an R^2^ value for goodness of fit, and the length of the indels. These should be similar for both the forward and reverse sequence for each sample, unless one primer returns a poor quality signal. Use only the values for high quality sequencing reads, where the R^2^ is above 0.8.
8. The total knockout percentage can be interpreted as the total efficiency score provided by the software, but this can be an underestimate and a more optimistic assessment would be to use 100% minus the percentage of wild-type sequence (0 indel) detected. This is further complicated by the presence of any in-frame indels observed, so inferring the real percentage of knockout using this method is difficult and confirmation by immunoblotting is recommended where reliable antibodies are available.

#### Assessing STAT3 deletion at the protein level

1. For verification of STAT3 protein deletion, the following controls and experimental samples are recommended: 1) non-transduced EpH4 cells, 2) cells targeted with the first STAT3 targeting guide, 3) cells targeted with a second STAT3 targeting guide and 4) cells targeted with a non-targeting control guide (or a neutral-targeting control guide).
2. For each cell line, prepare protein samples in RIPA buffer containing protease inhibitors. Prepare an immunoblot with 10 µg of protein from each sample using standard protocols.
3. Probe blots for total STAT3. For well-designed CRISPR guides, and when antibodies reliably detect only the protein of interest, we regularly see complete protein ablation using this method.

### Microscopic analysis of the cellular vesicular compartment in response to STAT3 activation

**Fig. 4:**
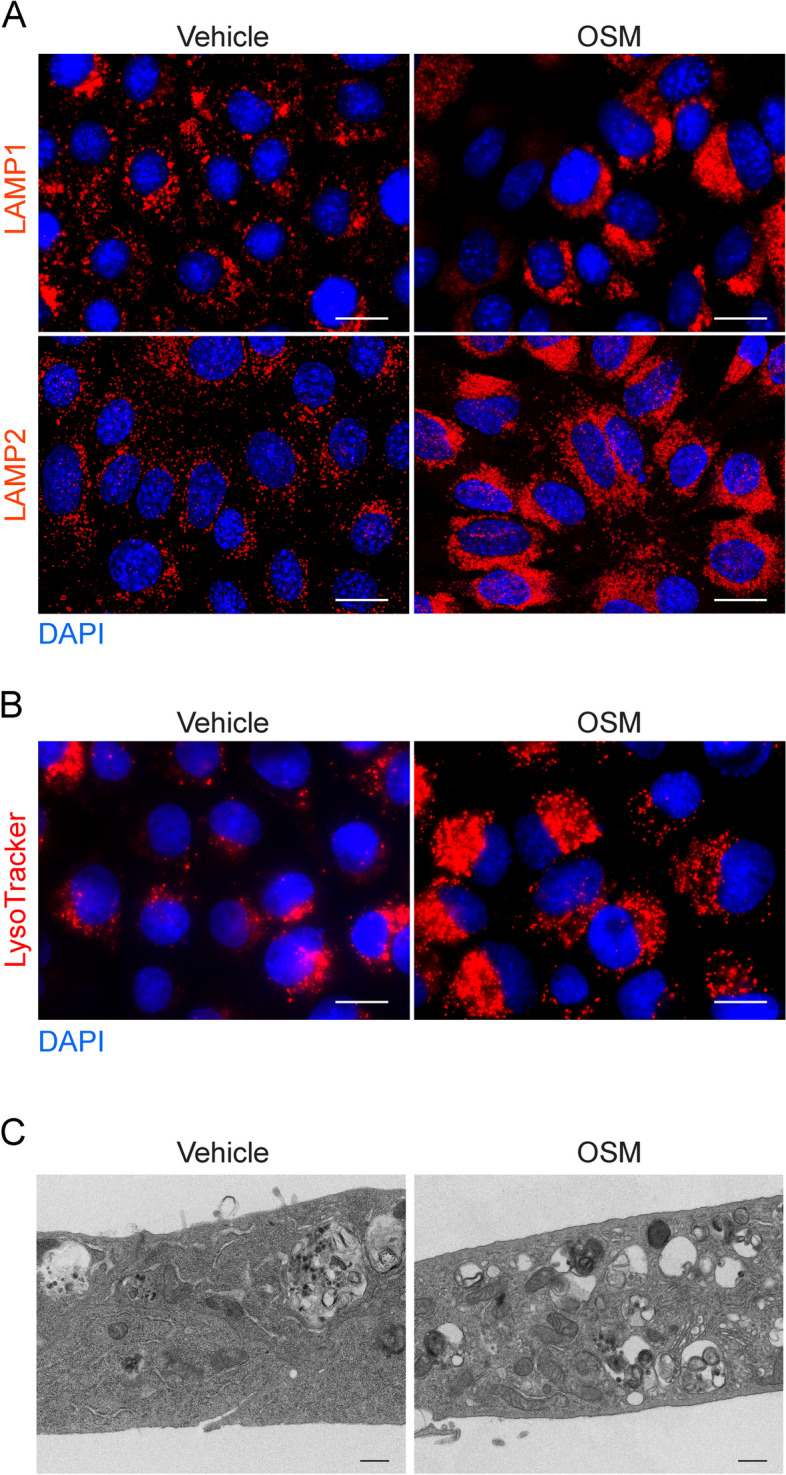
STAT3-mediated upregulation of the vesicular compartment in EpH4 cells. **A** Fluorescence microscopy of LAMP1 and LAMP2 immunostaining in EpH4 cells treated with OSM for 72 h show upregulation of the lysosomal compartment with STAT3 activation. Scale bars: 20 μm. **B** Fluorescence microscopy of LysoTracker® staining in EpH4 cells treated with OSM for 72 h show upregulation of the acidic compartment with STAT3 activation. Scale bars: 20 μm. **C** Transmission electron microscopy images of EpH4 cells showing an increased number of degradative vesicles after 72 h of OSM stimulation. Scale bars: 500 nm

Microscopic analysis of mammary gland tissues revealed that STAT3-mediated LM-PCD during mammary gland involution is characterised by the formation of large lysosomal-like vacuoles in mammary epithelial cells and increased cathepsin protease expression [8, 14]. This enhancement of the lysosomal system downstream of activated STAT3 signalling is also apparent when stimulating EpH4 cells with OSM [8, 15] (Fig. 4). In this section, we provide three protocols for microscopic visualisation of the vesicular/lysosomal compartment in response to STAT3 activation in cell lines.

#### Method 1: Immunofluorescence staining of the lysosomal compartment

1. Seed cells on glass coverslips or glass-bottom dishes at the desired confluency (*see* Note 11).
2. Next day, stimulate STAT3 activity in cultured cells e.g. using cytokines such as OSM (as described for EpH4 cells).
3. At the desired timepoint after STAT3 activation, remove media and wash cells in PBS.
4. Fix cells in 4% (v/v) paraformaldehyde in PBS for 5 min at 37 °C (*see* Note 12).
5. Wash cells 2 times in PBS and permeabilise by incubating in 0.5% (v/v) Triton X-100 in PBS for 10 min at room temperature (*see* Note 12).
6. Block cells in 10% (v/v) normal goat serum in PBS for 1 h at room temperature. Alternatively, 1% (w/v) BSA in PBS may be used.
7. Incubate cells with primary antibody diluted in PBS for 1 h at room temperature, or overnight at 4 °C in a humidified chamber. For example, a rat anti-LAMP2 antibody or rat anti-LAMP1 antibody can be used to stain lysosome membranes (see Fig. 4A for example images). Antibodies that label lysosomal cathepsin proteases (e.g. cathepsin B/D/L) may also be used.
8. Wash cells 3 × 5 min in PBS at room temperature. If using directly-conjugated primary antibodies, proceed directly to step 11.
9. Apply fluorescent-dye conjugated secondary antibodies diluted in PBS (1:500) and incubate in a humidified chamber for 1 h at room temperature. Protect samples from light from this point forth.
10. Wash cells 3 × 5 min in PBS at room temperature.
11. Apply DAPI solution and incubate for 15 min at room temperature to stain nuclei.
12. Remove DAPI solution and wash 3 × 5 min in PBS at room temperature.
13. If using glass coverslips, dry excess PBS and place face down onto a small drop of glycerol mounting solution (10–20 μl depending on the size of the coverslip) on a glass slide, taking care to avoid bubble formation. Secure the coverslip using nail varnish around coverslip edges. Store slides at 4 °C and protect from light until imaging.

#### Method 2: LysoTracker® staining of acidic organelles

1. Seed cells on glass coverslips or glass-bottom dishes at the desired confluency (*see* Note 11).
2. Next day, stimulate STAT3 activity in cultured cells e.g. using cytokines such as OSM (as described for EpH4 cells).
3. At the desired timepoint after STAT3 activation, replace medium with fresh growth medium containing LysoTracker® fluorescent dye (e.g. LysoTracker® Red DND-99, Fig. 4B) at a dilution of 1:10,000 to label acidic organelles (such as lysosomes).
4. Incubate cells in LysoTracker-containing medium for 30 min.
5. Remove medium and wash cells 2 × 5 min in PBS.
6. Fix cells in 4% (v/v) paraformaldehyde in PBS for 5 min at 37 °C.
7. Wash cells 2 × 5 min in PBS.
8. Apply DAPI solution and incubate for 15 min at room temperature to stain nuclei.
9. Remove DAPI solution and wash cells 3 × 5 min in PBS at room temperature.
10. If using glass coverslips, dry excess PBS and place face down onto a small drop of glycerol mounting solution (10–20 μl depending on the size of the coverslip) on a glass slide, taking care to avoid bubble formation. Secure the coverslip using nail varnish around coverslip edges and image as soon as possible after fixation (*see* Note 13).

#### Method 3: Transmission electron microscopy of the vesicular compartment

1. Seed cells in 10 cm tissue culture dishes at a confluency that will reach approximately 80–90% by the time the cells are harvested. A 10 cm dish is necessary as a large cell pellet is required to ensure enough material survives processing through to resin blocks (outlined below).
2. Next day, stimulate STAT3 activity in cultured cells e.g. using cytokines such as OSM (as described for EpH4 cells).
3. At the desired timepoint after STAT3 activation, remove media and wash cells in PBS.
4. Remove PBS and add fixative solution and incubate overnight at 4 °C. Perform all steps using fixative in a chemical fume hood with local exhaust ventilation.
5. Scrape cells in fixative solution and transfer to 1.5 ml tubes.
6. Pellet cells by centrifuging at 2000 × *g* for 10 min at 4 °C.
7. Wash cells once with sucrose solution.
8. Fix lipids by incubating in 2% (w/v) osmium tetroxide in water for 1 h with rotation (very hazardous material, *see* Note 4).
9. Wash cells once with sucrose solution.
10. De-hydrate cells by serially incubating in increasing concentrations of ethanol (70%, 90%, 95% and 100%) for 30 min per concentration.
11. Treat cells with propylene oxide for 30 min (very hazardous material, see Note 4).
12. Incubate cells in a 1:1 solution of 100% propylene oxide:100% resin for 1 h, followed by a 2-day incubation in pure resin to ensure efficient infiltration.
13. Embed cells in embedding moulds containing fresh resin and polymerise at 70 °C for 24 h.
14. Cut sections at a thickness of 80 nm using an ultramicrotome and place on copper mesh grids.
15. Stain sections by placing grid section-side down on a drop of 4% (w/v) aqueous uranyl acetate (very hazardous material, see Note 4) for 8 min and wash with water by dipping grids into three beakers of water 20 times each. Dry the grid using filter paper.
16. Stain sections with Reynolds’ lead citrate stain [19] by placing the grid section side down in a drop of Reynolds’ lead citrate stain inside a petri dish that also contains sodium hydroxide pellets that are not touching the stain solution (*see* Note 14). Stain for 8 min and wash with water as above.
17. Dry the grid using blotting paper and store in a dry place until analysis by transmission electron microscopy (example representative images shown in Fig. 4C).

## Notes

1. The EpH4 cell line is available from Sigma (product code: SCC284).
2. For assays focused on measuring the activity of cathepsin proteases, do not use protease inhibitors that block cysteine proteases. 1 mM Pefabloc (Fluka), which specifically inhibits the activity of serine proteases, can be used instead. If no cathepsin activity is to be measured, cOmplete Protease Inhibitor Cocktail can be used.
3. LysoTracker® probes are fluorescent dyes (available in a variety of fluorescent colours) that enable specific labelling and tracking of acidic organelles in live cells, including lysosomes. They are compatible with aldehyde-based fixatives.
4. Hazardous and extremely toxic material—please seek appropriate training and refer to local and national guidelines. Strictly adhere to safety guidelines in the accompanying materials safety data sheet.
5. EpH4 cells are more resistant to OSM-induced cell death if overconfluent at the time of stimulation. Culturing cells in 1% (v/v) FCS containing growth medium prevents them from becoming overgrown during study timeframes.
6. For lysosomal proteomic or large-scale leakage studies, we typically seed 3—5 × 15 cm plates per condition to ensure sufficient material for these assays.
7. The heavily glycosylated nature of many lysosomal membrane proteins can interfere with mass spectrometry analysis. Thus, we recommend pre-treating lysosomal membrane fractions with PNGaseF (an amidase that removes N-linked oligosaccharides from glycoproteins) prior to proteomic analysis, which substantially improves the representation and identification of lysosomal membrane proteins by LC–MS/MS [15].
8. To minimise the risk of off-target effects from the constitutive SpCas9 and sgRNA expression, cells should be analysed at low passage number. Prepare a large batch (20–30 vials) of cryopreserved cells at passage 3 post-lentiviral transduction and discard cells that reach 10 passages post-transduction.
9. Perform all lentiviral work under BSL2 or enhanced BSL2 containment according to your institution’s guidance and risk assessments. All disposable laboratory materials and waste must be decontaminated by autoclaving or chemical disinfection prior to disposal into the biohazardous waste stream.
10. The TIDE analysis website has an in-depth description of how to interpret the data produced, and how to troubleshoot if error messages are encountered. Default parameters typically give good results if high quality sequencing traces are obtained. However, increasing the indel size range to 25–50 can sometimes reveal larger indels that would be missed with the default value of 10.
11. Do not allow cells to become overconfluent as changes to the vesicular compartment are harder to discern in densely packed cells.
12. Alternatively, cells may be fixed and permeabilised using 100% methanol (for 5 min at room temperature) for downstream vesicular immunostaining with comparable results.
13. While LysoTracker® fluorescent dyes are well retained after aldehyde-based cell fixation, it is advisable to image immediately to minimise risk of signal loss.
14. Sodium hydroxide pellets remove atmospheric carbon dioxide that reacts with the lead stain [26].
